# Plaque modification of severely calcified coronary lesions via orbital atherectomy: Single‐center observations from a complex Veterans Affairs cohort

**DOI:** 10.1002/hsr2.99

**Published:** 2018-10-27

**Authors:** Rupak Desai, Omer Mirza, Brad J. Martinsen, Gautam Kumar

**Affiliations:** ^1^ Division of Cardiology Emory University and Atlanta Veterans Affairs Medical Center Atlanta GA USA; ^2^ Department of Clinical and Scientific Affairs, Cardiovascular Systems, Inc. St. Paul MN USA

**Keywords:** chocolate focal force angioplasty, coronary artery calcification, coronary artery disease, orbital atherectomy, percutaneous coronary intervention, complex PCI

## Abstract

**Background:**

Orbital atherectomy (OA) is a known alternative to other atherectomy devices. However, some complex patient demographics (eg, left ventricular ejection fraction <25%) were excluded from the first‐in‐human trial (ORBIT I) and the pivotal FDA device approval trial (ORBIT II) which evaluated the safety and efficacy of OA in severely calcified de novo coronary lesions. This single‐operator cohort study aimed to examine the impact of OA on a real‐world complex Veterans Affairs patient subset.

**Methods:**

Retrospective analysis was completed on 40 consecutive patients with severely calcified coronary lesions who underwent OA prior to drug‐eluting stent placement at the Atlanta Veterans Affairs Medical Center from January 2015 to June 2017.

**Results:**

Orbital atherectomy plus drug‐eluting stent placement was successful in all 40 cases. Chocolate focal force balloon angioplasty was the most commonly used post‐atherectomy balloon (*N* = 34, 85%). Few complications were observed, including one case (2.5%) of perforation and one case (2.5%) of no‐reflow. Neither acute stent thrombosis nor emergent coronary artery bypass grafting was observed. The intravascular ultrasound (IVUS)‐determined median [IQR] pre‐procedure minimum lumen area and post‐procedure minimum stent area (MSA) were 2.8 [2.2, 3.0] mm^2^ and 8.7 [7.7, 10.0] mm^2^, respectively (*P* < 0.0001, Mann‐Whitney test). Major adverse cardiovascular events, including all‐cause mortality, at 30 days and at a median [IQR] follow‐up of 197.5 [35.5, 461.3] days, was 5% and 10%, respectively. During that period, one target vessel revascularization (2.5%) was observed.

**Conclusions:**

This study indicates that OA is a useful tool in performing high‐risk percutaneous coronary intervention effectively in VA patients with severely calcified coronary lesions. OA plaque modification in combination with a high utilization rate of IVUS and Chocolate focal force angioplasty facilitates stent delivery and optimal stent expansion, resulting in a large MSA.

## 1 INTRODUCTION

A steadfast antagonist to the interventional cardiologist, dense coronary calcifications of atheromatous plaque provide a major hindrance to successful revascularization. While utilization of intravascular ultrasound (IVUS) can improve detection of calcification compared with fluoroscopy alone (73% versus 38%, respectively),^1^ the rigid plaque can complicate adequate stent deployment. Examples include increased risk of in‐stent restenosis or stent thrombosis with asymmetric stent expansion,^2^, ^3^, ^4^, ^5^ while aggressive balloon dilation in noncompliant anatomy can result in coronary dissection.^6^ Thus, pretreatment interventions focus on decreasing the degree of calcifications to facilitate adequate angioplasty. The Diamondback 360 coronary orbital atherectomy (OA) system (Cardiovascular Systems, Inc. [CSI], St Paul, MN) is a proven asset as an alternative to rotational atherectomy (RA) for the treatment of these heavily calcified coronary lesions.^7^, ^8^, ^9^, ^10^, ^11^ The purpose of this study was to retrospectively analyze the safety and effectiveness of OA for the treatment of high‐risk VA patients with severely calcified coronary lesions. We present here the acute procedural, 30‐day, and 6.5‐month median follow‐up outcomes.

## 2 MATERIALS AND METHODS

### 2.1 Atherectomy device description

The Diamondback 360 coronary OA system (Cardiovascular Systems, Inc. [CSI], St Paul, MN) was utilized in all cases analyzed. As previously reviewed, the mechanism of this device has been shown to be useful in the treatment of severely calcified, noncompliant coronary lesions prior to appropriate percutaneous transluminal coronary angioplasty and adequate stent deployment.^11^

### 2.2 Study population

In this single center study, an IRB approved (IRB00094268) retrospective chart review of all patients who underwent coronary angiography and angioplasty from January 2015 to June 2017 at Atlanta Veterans Affairs Medical Center was undertaken. This research was eligible for expedited review under 45 CFR.46.110 and/or 21 CFR 56.110 because it posed minimal risk and fit the regulatory categories of research #5 as set forth in the Federal Register. The Emory IRB reviewed it by expedited process on 2.05.2017 and granted approval effective from 2.05.2017 through 2.04.2018 (a waiver of informed consent and a complete HIPPAA waiver were granted). Inclusion criteria required patients to be at least 18 years old and less than 95 years old, undergoing appropriate coronary angiography per clinical indication, to have severely calcified coronary plaque determined by fluoroscopic imaging^1^ or IVUS,^1^ flow limiting lesion defined as lesion >70% but <100% of vessel diameter, and most notably, utilization of OA to assist prior to coronary revascularization. All lesions were functionally significant as measured by invasive methods (fractional flow reserve or instantaneous wave‐free ratio) or non‐invasive methods (nuclear stress test).

### 2.3 Data extraction and statistical analysis

A comprehensive chart review of clinical records was completed to extract patient demographics, indication for coronary angiography/intervention, lesion and target vessel characteristics, as well as acute/procedural and outcome results. The IBM Statistical Package for the Social Sciences (SPSS) Statistics 22.0 (IBM Corp., Armonk, NY) and Excel 2016 (Microsoft, Redmond, WA) were utilized to perform the analyses. Normality was evaluated by the Shapiro‐Wilk test in SPSS 22 (*P* > 0.05 normal distribution). Medians are presented for non‐normally distributed data.

### 2.4 Primary safety and efficacy endpoint

Clinical documentation was abstracted and analyzed to determine the coronary revascularization success rate, procedural acute angiographic complication rates, and 30‐day and 6.5‐month median follow‐up major adverse cardiac and cerebrovascular event rates (MACCE; defined as myocardial infarction requiring cardiac catheterization or intervention, stroke, target vessel revascularization, and death).

## 3 RESULTS

### 3.1 Baseline subject demographics

Forty subjects met all of the inclusion criteria for this retrospective analysis. The baseline subject demographics are shown in Table 1. All subjects were male, with a median [IQR] age of 68.5 [65.5, 70.0] years, and the majority exhibited high rates of diabetes mellitus, hypertension, hyperlipidemia, and smoking status. This subject cohort analysis included 13 (32.5%) patients who had baseline chronic kidney disease (CKD; defined as Cr > 1.5 mg/dL) and six (15%) cases with Cr > 2.5 mg/dL, of which five cases (12.5% of the total number of patients) were already on hemodialysis. This complex cohort also exhibited left ventricular systolic dysfunction—three patients had a left ventricular ejection fraction ≤25%. Lastly, the most common indication for percutaneous coronary intervention (PCI) was unstable angina followed by elective and myocardial infarction.

**Table 1:** Baseline subject demographics

	Total (*N* = 40)
Age (years)	68.5 [65.5, 70.0]
Male	40 (100)
Co‐morbidities	
Hypertension	38 (95)
Diabetes	22 (55)
Hyperlipidemia	36 (90)
Smoking	24 (60)
CKD (creatinine ≥1.5 mg/dL)	13 (32.5)
CKD (creatinine ≥2.5 mg/dL)	6 (15)
Dialysis	5 (12.5)
Previous history of	
MI	15 (37.5)
PCI	15 (37.5)
CABG	9 (22.5)
Indication for PCI	
Unstable angina	22 (55)
MI	7 (17.5)
Elective	9 (22.5)
Other (ischemic cardiomyopathy)	2 (5)
Left ventricular ejection fraction (%)	57.5 [50.0, 61.3]
Left ventricular systolic dysfunction (<40%)	6 (15)
Left ventricular systolic dysfunction (≤25%)	3 (7.5)

Values are *n* (%) or median [interquartile range Q1, Q3].

Abbreviations: CABG, coronary artery bypass graft; CKD, chronic kidney disease; MI, myocardial infarction; PCI, percutaneous coronary intervention.

### 3.2 Index lesion/vessel characteristics and procedural outcomes

As shown in Table 2, trans‐radial was the most common access site. 17 cases (42.5%) utilized 7.5 Fr sheathless guide catheters (Asahi Intecc USA Inc, Santa Ana, CA) and the others used conventional 6 or 7 Fr guide catheters. The majority of the lesions treated were located in the left anterior descending and left circumflex vessels. The median [IQR] pre‐procedural lesion stenosis was 80% [70%, 90%], and fractional flow reserve and instantaneous wave‐free ratio measurements prior to treatment indicated hemodynamically significant stenosis in 24 of the cases. Three subjects required multi‐vessel PCI. In addition, an Impella (Abiomed, Danvers, MA) hemodynamic support device was utilized in eight cases. All cases involved OA, and the median [IQR] number of OA passes was 5.5 [5.0, 7.5] per case, with high‐speed passes (120 000 rpm) used in 60% (*N* = 24) of cases. The Chocolate focal force balloon (Trireme Medical, Inc., Pleasanton, CA) was the most commonly used post‐atherectomy angioplasty device, resulting in low median maximum inflation pressures. Post atherectomy and angioplasty, all cases resulted in successful drug‐eluting stent placement and the median [IQR] number of drug‐eluting stent placed per case was 1 [1, 2], with a median post‐procedural stenosis of 0% (mean ± SD 0.2 ± 1.1%). Lastly, IVUS analysis (retrospective online analysis—non core‐lab adjudicated) indicated a median [IRQ] pre‐procedure minimum lumen area and post‐procedure minimum stent area (MSA) of 2.8 [2.2, 3.0] mm^2^ and 8.7 [7.7, 10.0] mm^2^, respectively (*P* < 0.0001, Mann‐Whitney test).

**Table 2:** Index lesion/vessel characteristics and procedural outcomes

	Total (*N* = 40)
Access	
Radial	24 (60)
Femoral	16 (40)
Guide catheter size	
6 Fr	10 (25)
7 Fr	13 (32.5)
7.5 Fr	17 (42.5)
Multi‐vessel PCI	3 (7.5)
Total vessels treated per case	1 [1, 1]
Vessels treated	
Left main	6 (15)
Left anterior descending	25 (62.5)
Left circumflex	8 (22.2)
Diagonal	1 (2.8)
Obtuse marginal	2 (5.6)
Right coronary artery	5 (13.9)
Quantitative coronary angiography (QCA)	
Reference vessel diameter (mm)	3.5 [3.3, 3.8]
Lesion length (mm)	32.5 [24.0, 36.0]
Pre‐procedural MLD (mm)	0.8 [0.8, 1.0]
Post‐procedural MLD (mm)	3.0 [2.8, 3.0]
Pre‐procedural stenosis (%)	80 [70, 90]
Post‐procedural stenosis (%)	0 [0, 0]
Intravascular ultrasound (IVUS)	
Pre‐procedure IVUS	32 (80)
Post‐procedure IVUS	38 (95)
Pre‐procedure MLA (mm^2^)	2.8 [2.2, 3.0]
Post‐procedure MSA (mm^2^)	8.7 [7.7, 10.0]
FFR/iFR measured	24 (60)
FFR pre‐procedure	0.70 [0.65, 0.75]
iFR pre‐procedure	0.82 [0.81, 0.85]
OA procedure	
Total number of passes per case	5.5 [5.0, 7.5]
At low speed 80 000 rpm	4.0 [3.0, 4.0]
At high speed 120 000 rpm	2.0 [1.0, 3.0]
High speed pass used	24 (60)
Post‐atherectomy balloon	
Chocolate focal force	34 (85)
Semi‐compliant POBA	0 (0)
Non‐compliant POBA	6 (15)
Maximum balloon pressure (atm)	16 [14, 18]
Drug‐eluting stent	40 (100)
Total stents used per case	1 [1, 2]
Maximum stent pressure (atm)	14 [14, 15]
Volume of contrast (mL)	152.5 [120.0, 200.0]
Total fluoroscopy time (min)	33.3 [27.3, 37.9]
Impella support device used	8 (20)
Dual antiplatelet therapy after catheterization	40 (100)
Successful stent placement	40 (100)

Values are *n* (%) or median [interquartile range Q1, Q3].

Abbreviations: FFR, fractional flow reserve; iFR, instant wave‐free ratio version of FFR; MLA, minimal lumen area; MLD, minimum lumen diameter; MSA, minimal stent area; POBA, plain‐old‐balloon angioplasty; OA, orbital atherectomy.

### 3.3 Angiographic complication rates and MACCE outcomes

The angiographic and MACCE outcomes are shown in Table 3. Briefly, there was no evidence of in‐stent thrombosis or significant coronary spasm in any of the cases. However, minor rates of coronary perforation (*N* = 1, 2.5%) and no‐reflow (*N* = 1, 2.5%) occurred. None of the subjects experienced MACCE at index (in‐hospital). The 30‐day MACCE rate was 5%, and the two events were death (both cases were high risk with Impella assistance: one had coronary perforation which was successfully sealed and later had probable stent thrombosis due to suspected DAPT interruption, and the other died of multi‐organ failure). The median [IQR] time to a MACCE and/or the last known clinic follow‐up visit was 197.5 [35.5, 461.3] days (~6.5 months), resulting in four (10%) subjects with a MACCE. Briefly, one additional death (due to septic shock) and one target vessel revascularization occurred post 30 days.

**Table 3:** Angiographic complication rates and MACCE outcomes

	Total (*N* = 40)
Angiographic complications	2 (5.0)
Perforation	1 (2.5)
Dissection	0 (0.0)
No‐reflow	1 (2.5)
MACCE 30 days	2 (5.0)
Death	2 (5.0)
MI (requiring intervention)	0 (0.0)
Stroke	0 (0.0)
TVR	0 (0.0)
Stent thrombosis 30 days	0 (0.0)
MACCE 6.5 months (median 197.5 days)	4 (10)
Death	3 (7.5)
MI (requiring intervention)	0 (0.0)
Stroke	0 (0.0)
TVR	1 (2.5)
Stent thrombosis 6.5 months	0 (0.0)

Values are *n* (%).

Abbreviations: MACCE, major adverse cardiac and cerebrovascular events; MI, myocardial infarction; TVR, target vessel revascularization.

## 4 DISCUSSION

This single‐center retrospective analysis of a Veterans Affairs cohort with severe coronary artery calcification revealed high efficacy and safety rates despite the complex disease state noted in these patients. In addition, the high utilization rate of IVUS and Chocolate focal force angioplasty in combination with OA resulted in a large MSA.

The optimal results presented here are likely due to the unique mechanism of action of OA and Chocolate balloon angioplasty. As previously reviewed, OA has an orbital and differential sanding mechanism that reduces plaque burden, creating vessel compliance while minimizing damage to the medial layer of the vessel.^9^, ^10^, ^11^ The Chocolate balloon consists of a mounted nitinol constraining structure specifically designed for uniform, controlled inflation and rapid deflation, resulting in atraumatic dilatation without the need for cutting or scoring. Because of its novel design, it can enable focused force angioplasty from its “pillows” while allowing plaque channeling through its “grooves.”^12^

In addition to the OA intraluminal plaque modification ability, it is also known that the OA crown creates pulsatile forces that result in fissures of the medial calcification.^13^ Unlike intimal calcification, medial calcification results from elastin fiber mineralization and increased production of osteochondrogenic proteins, something typically seen in diabetes mellitus and CKD.^10^ Thus, we hypothesize that there may be pulsatile forces that indirectly impact deeper calcification in the coronary vessel wall, similar to what has been shown with the peripheral OA device.^13^ These medial fissures may be further expanded by the pillows of the Chocolate balloon resulting in one mechanism to account for the numerically larger average MSA (mm^2^) seen in this study (mean ± SD 8.9 ± 1.2; median [IQR] 8.7 [7.7, 10.0]) versus a previous OA (6.43 ± 1.86) and RA (5.40 ± 1.70) imaging study.^14^ These results expand on a previously published case example showing the synergy between OA and Chocolate balloon.^12^ In addition, these results have important implications as it has previously been shown that greater post‐intervention MSA is a predictor of better outcomes and patency.^14^, ^15^, ^16^

Lastly, the angiographic complication and MACCE outcome rates seen in this VA retrospective analysis are similar to past OA clinical studies (ORBIT I, ORBIT II, and other multicenter studies), which have been extensively reviewed by others and compared with other atherectomy devices.^8^, ^9^, ^10^, ^11^ In particular, patients with known CKD (including those on hemodialysis; excluded from ORBIT I), who are at higher risk for development of calcified lesions, as well as those with severe heart failure (left ventricular ejection fraction <25%; excluded from ORBIT I and II), were included in this analysis. Thus, the evidence presented here is crucial in providing a signal that OA may benefit complex patients, especially those at higher risk for development of severely calcified lesions and subsequent MACCE.

### 4.1 Study limitations

This was a small, non‐randomized, retrospective study with minimum of 30‐day follow‐up (median [IQR] 197.5 [35.5, 461.3] days follow‐up). Neither comparison with RA nor PCI without atherectomy was performed. Periprocedural cardiovascular biomarkers were not acquired for all patients; thus, myocardial infarction rates could have been underdiagnosed. All procedures were performed by a single operator and, thus, results may not be generalizable to all medical centers and operators considering a difference in training and expertise. Randomized trials are needed to clearly determine the ideal treatment strategy for severely calcified coronary lesions. The ECLIPSE randomized coronary orbital atherectomy trial (2000 subjects) is now underway to determine the ideal strategy (Clinicaltrials.gov identifier NCT03108456).^11^

## 5 CONCLUSION

Orbital atherectomy is a useful tool in performing high‐risk PCI effectively in VA patients with severely calcified coronary lesions. OA plaque modification in combination with IVUS and Chocolate focal force angioplasty facilitates stent delivery and optimal stent expansion, resulting in a large minimum stent area.

## CONFLICTS OF INTEREST

R.D. and O.M. have no declarations of interest. B.J.M. is an employee of CSI. G.K. has a *pro bono* consulting agreement with CSI. These conflicts did not affect the study design, conduct, or reporting.

## AUTHOR CONTRIBUTION

Conceptualization: Rupak Desai, Omer Mirza, Brad Martinsen, Gautam Kumar

Data Curation: Rupak DesaiFormal Analysis: Rupak Desai

Investigation: Rupak Desai, Omer Mirza, Gautam Kumar

Methodology: Rupak Desai, Omer Mirza, Brad Martinsen, Gautam Kumar

Project Administration: Gautam Kumar

Resources: Gautam Kumar

Supervision: Gautam Kumar

Validation: Rupak Desai

Visualization: Brad Martinsen

Writing‐Original Draft Preparation: Rupak Desai, Omer Mirza, Brad Martinsen, Gautam Kumar

Writing‐Review and Editing: Rupak Desai, Brad Martinsen, Gautam Kumar
